# The Use and Creation of Photographic Imagery in Global Health: Actionable Steps Towards Decolonization by Academic Institutions

**DOI:** 10.5334/aogh.4847

**Published:** 2025-09-03

**Authors:** Meagan Harrison, Anna Kalbarczyk, Bareng Aletta Sanny Nonyane

**Affiliations:** 1 Department of International Health, Johns Hopkins Bloomberg School of Public Health, Baltimore, Maryland, USA

**Keywords:** ethical image use, visual representation in global health, decolonizing academia, visual storytelling, image ethics, higher education, global health

## Abstract

*Background:* Photographic imagery holds profound power in shaping narratives, identities, and perceptions in global health education. Historically, visual representation used in global health has perpetuated colonial hierarchies, reinforcing inequities and marginalizing the voices and lived realities of the communities they depict. These images can inadvertently sustain harmful stereotypes and distort the complexity of global health challenges.

*Findings:* This paper explores the ethical imperative of decolonizing photographic imagery within academic global health, proposing a comprehensive multi-level framework for change targeting institutions, faculty, and students. At the institutional level, strategies include developing formal ethical image-use policies, establishing accountability structures, and providing ongoing training to center principles of informed consent, dignity, and cultural context in image selection and use. Faculty have a critical role in modeling ethical practices by selecting imagery in research outputs and teaching materials, integrating visual ethics into curricula, and fostering classroom dialogue that encourages critical reflection on representation and power dynamics. Educators can actively engage students by empowering them to contribute their own experiences, thereby reshaping dominant visual narratives. Collaboration with community partners in co-creating authentic and respectful images is essential, alongside mechanisms for continuous evaluation and accountability to sustain ethical standards over time.

*Recommendations:* We recommend that academic institutions adopt institution-wide ethical image-use policies, offer training programs for faculty and students, and develop centralized image repositories that include culturally appropriate and consented visuals. Faculty should integrate ethical image practices into research and pedagogy, while creating spaces for students to reflect on diverse perspectives. Building meaningful, ongoing partnerships with community stakeholders is crucial to ensuring that images represent the diversity and dignity of global health realities.

*Conclusions:* By advancing a culture of ethical reflexivity and accountability around photographic imagery, academic institutions can dismantle colonial visual legacies and foster more equitable, inclusive, and humanizing global health education and practice.

## Background

In recent years, the discourse surrounding decolonization efforts in global health has gained significant traction, both within academic circles and in broader public discourse [1–3]. Theoretically, decolonizing requires a comprehensive and multifaceted approach that breaks down current systems and rebuilds them from the ground up, but some have questioned whether it is possible to see a total and simultaneous overhaul of global health as we know it now [4]. Regardless of whether a complete overhaul is needed or appropriate, it is important that global health actors at every level aim to take steps, no matter how small they may seem, towards decolonization in their own spheres of influence.

One of the ways that global health academic institutions can actively, systematically, and immediately contribute to the decolonizing agenda is by addressing the use and creation of photographic imagery in their global health programs. Images remain a powerful vehicle for shaping perceptions and narratives and continue to be influenced by colonial histories and contemporary power dynamics [5]. Addressing the ways in which images are employed and produced holds particular significance due to their powerful role in shaping perceptions and narratives, and ultimately, policies and actions.

In this paper, we explore the nexus between global health decolonization and photographic imagery in the context of global health academic institutions. Building on existing frameworks [6] for how to decolonize global health photography, we apply the principles and offer actionable steps that academic institutions can undertake to interrogate the ethical dimensions of how images are created and used in their research, teaching, and communications domains.

## Findings

### Global health and ethical imagery

Much has been written about the ethical challenges related to photographic images, specifically related to photography’s interconnectedness with colonialism [6, 7]. Historically, photography has centered the white experience as “civilized,” othering people of color and reinforcing colonial ideals such as white saviorism, orientalism, and exploitation. Recently, even more scholarship has shed light on the longstanding pervasive nature of problematic imagery, specifically within the field of global health [5, 6].

A comprehensive review conducted by Charani et al. highlights the intersection of global health and photographic imagery. Their analysis illuminates the complex patterned dynamics underlying image use, from the perpetuation of colonial legacies to the reinforcement of power differentials and stereotypes. Harmful images can portray tropes such as malnourished children or impoverished groups from less‑resourced communities with the intention of evoking compassion and raising awareness and money for programs, but in reality are “generating pity rather than empathy, demeaning rather than empowering, and commercializing rather than representing the featured groups” [6].

The impact of imagery on perceptions and narratives within global health discourse cannot be overstated. Images possess the unique ability to evoke emotions, shape attitudes, and influence decision‑making processes. Used ethically, they can humanize complex issues; used poorly, they can perpetuate victimhood narratives, harmful stereotypes, and mistrust, undermining efforts to promote collaboration and sustainable development [6, 8].

Charani et al.’s review facilitated the identification of a framework of core concepts that should be upheld when capturing and distributing images in the global health context. According to their framework, images should be “relevant to the topic, respect[ful] of the integrity of all individuals depicted . . . accompanied by evidence of consent, and . . . equitable in representation” [6]. This framework promotes accuracy, authenticity, and transparency in representing diverse experiences and perspectives within global health contexts.

Not only can the content of unethical global health images be harmful, but the very process of how a photograph is made has traditionally reflected the colonialism that the resulting image perpetuates [8]. Typically, an outsider, usually white, travels to a community in a less‑resourced setting and takes photographs, often with questionable or nonexistent consent practices and little regard for the cultural perceptions of photography or the experience of the people who will be the subjects of their images. The photographer takes what they intended to acquire and departs, with no trust, input, follow‑up, or accountability to the community for what is depicted or how the images will be used [9, 10]. The Charani et al. framework challenges this pattern, emphasizing the importance of maintaining ethical integrity throughout both the image creation and image utilization processes.

## Recommendations

### What can academic institutions do?

Academic institutions can play a pivotal role in advancing the agenda of decolonizing photographic imagery in global health education, research, and practice. They serve as institutional hubs for academic and professional development, with the capacity to shape organizational policies, practices, and culture. By leveraging their institutional resources, expertise, and influence, academic institutions have the capacity to effect meaningful change and promote ethical practices in image use and creation. Here, we outline actionable steps that institutions can undertake at the institution‑wide, research and classroom levels to align with the principles of consent, integrity, relevance, and equitable representation to foster a culture of inclusion and respect the way its members use and create images.

### Review of current image‑use practices

The first step towards decolonizing photographic imagery within university settings should involve conducting a comprehensive review of current image‑use practices within the institution. This entails examining existing images featured in images used across campuses in physical buildings, course materials, communications, and repositories to assess their alignment with ethical photography standards. Images that fail to meet these standards should be promptly removed and excluded from future use.

Institutions should also audit communications materials (brochures, websites, social media, fundraising campaigns) to ensure they feature diverse and inclusive imagery. Aligning promotional efforts with ethical principles strengthens credibility and reinforces a culture of equity and respect.

### Institutional‑level recommendations

At the institutional level, it is important to develop centralized supportive ethical imagery policies and provide oversight for ensuring consistent implementation. As part of their image‑use strategy, institutions should also spend time to identify and evaluate what they want to portray through the imagery they use. Images can be used to show the work done by the institution, to highlight the work of partners, for art, for education, and for storytelling, among other purposes. Upon agreeing to such purposes and policies, institutions can begin to assess their current image‑use practices. Once an assessment is done, efforts should be made to create infrastructure to protect and support appropriate image use.

#### Policies and oversight

Academic institutions must provide intentional support in the form of policy and oversight to sustain ongoing efforts towards decolonizing photographic imagery. This includes establishing guidelines and policies governing image use, allocating resources for training programs, and fostering a culture of accountability and transparency. Ideally, the institution would adopt a criteria for what constitutes ethical imagery, established by a representative group of partners and stakeholders, taking into consideration global photography ethics [6].

#### Supportive infrastructure

*Establishment of guidelines on ethical images:* After a review of current image‑use practices is conducted, institutions should create ethical image guidelines with input from faculty, students, and in‑country partners. Guidelines must be clear, actionable, and widely shared.

*Training and capacity building:* All affiliates should be trained in ethical image guidelines. Training should cover consent, cultural sensitivity, and how to deconstruct colonial narratives embedded in visual representations. The goal of these trainings would be for individuals to use critical thinking skills, compassion, and empathy when making and selecting images for their communications uses.

*Training for students completing global health experiences abroad:* Institutions that facilitate student practicums or field experiences in international settings should provide training on ethical image use and creation prior to departure. By equipping students with the necessary knowledge and skills, institutions can ensure that student practicums contribute to positive, respectful, and ethical engagement with global health issues.

*Establishment of a repository of ethical images:* Institutions could consider establishing a repository of ethical images that meet standards of consent, integrity, and relevance [6]. This repository would serve as a centralized resource for curated images available for publications, websites, teaching materials, and social media.

Importantly, images included in the repository should not only meet ethical content standards [6] but also contextual considerations, reflecting the diverse contexts and experiences within global health settings. In addition to being screened for ethical suitability, all included images would need to meet excellence in photography as well as image quality standards. A centralized repository helps safeguard against the use of harmful or inappropriate images and streamlines access to approved visuals.

Maintaining a repository requires institutional investment, including a team to curate, approve, and update images.

*Developing a repository in collaboration with local partners and communities:* One way to add images to a repository is through the creation of new images, universities should prioritize collaboration with local partners and communities, ensuring their active participation and representation in the image‑making process. Participatory photography initiatives such as Photovoice [11–13] offer a method platform for communities to share their perspectives and narratives through photography, empowering them to shape the visual discourse surrounding global health issues. By engaging in this meaningful co‑creation processes, academic institutions can foster mutual respect, trust, and reciprocity with local stakeholders and use the ethically created images for their repository, and ultimately in the media that comes out of the university.

Another way to add to the repository is to invite affiliates and local partners to submit photographs they have taken. As an example, the Knowledge for Health K4Health’s Photoshare project was based at Johns Hopkins University Bloomberg School of Public Health Center for Communication Programs and helped international nonprofit organizations communicate about health and development issues through photography. Photoshare hosted an annual photography contest and collected over 35,000 health and development images that were free for nonprofit and educational use [14]. A similar model could be followed for a repository initiative, encouraging local partners to submit photographs from their projects. Excellent photographs that meet ethical standards could be added as pre‑approved images.

Existing images may also be curated from the Internet. In this instance, trained staff could search for open‑source images that meet the required criteria and add them to the institution’s repository.

*Addressing repository challenges:* A more intensive ethical image‑use policy would require that university affiliates only use pre‑approved images in their publications, reports, websites, courses, and other materials. A potential issue that could arise with this type of mandate is if affiliates want to use images that are not already included in the repository. There are several ways to manage this challenge. One solution is to invest in hiring personnel to oversee all repository activities in the institution‑wide communications department, who would be trained to review and approve images according to the established criteria, adding them to the repository. Affiliates would then be able to submit their desired images for approval and addition to the repository before use. A more flexible policy could require affiliates to evaluate their desired images according to the established criteria and sign an honor pledge attesting to their evaluation before using an image that is not already pre‑approved.

### Creating a culture of ethical image use

Regular training should be integrated systematically to train all university affiliates on ethical image use so that they can make appropriate decisions for image use on their own. The hope is that eventually, institutions build a culture in which their affiliates not only make ethical decisions with images, but that they ultimately consider others compassionately and treat others with dignity and kindness as a rule for all their decisions.

### Implementation at the faculty research level

While it is important for institutions to lead with supportive policies and infrastructure development at baseline, it is critical that faculty members integrate these tools into their day‑to‑day operations. Faculty play a crucial role in shaping research agendas, methodologies, outputs, and culture within academic institutions. Here, we outline specific actions that faculty members can take to uphold the core principles of ethical image use in their research and teaching endeavors.

### Faculty training on ethical image use and creation

Academic institutions should integrate ethical image‑use principles into research and teaching practices for faculty. Research faculty and staff should receive training on ethical image use and creation, ensuring that images featured in reports, publications, and websites uphold the highest standards of integrity and respect outlined in the institution’s ethical image criteria policy. By equipping themselves with the necessary knowledge and skills, faculty members can serve as role models and advocates for ethical image use within their research and teaching communities.

### Local team‑led development of ethical image guidelines for visitors to in‑country sites

In addition to training the university community on ethical image guidelines before they travel, it is also important that university‑affiliated research and practice sites abroad establish their own guidelines for image creation and use in their organizations and share them with visitors to their sites. Local teams are best positioned to define what is appropriate in their context, drawing on their nuanced understanding of community norms and sensitivities around images. Faculty leaders such as principal investigators can play a facilitative role by offering support and resources as needed while learning from the lived experiences and perspectives of their in‑country colleagues. Institutions can further support this process by co‑developing adaptable training materials and templates in partnership with local teams, ensuring that guidelines are context‑specific and co‑owned. By centering the leadership and expertise of local teams and by fostering open, ongoing communication, research collaborations can more effectively prevent unintentional harm and promote respectful, ethical engagement with all communities involved.

### Use of ethical imagery in reports, publications, and online presence

Similarly to how universities should review the current state of image use at the institution level, faculty members should assess the images they are currently using in their research and practice programs. Faculty should prioritize the use of ethical imagery in their research reports, publications, and dissemination materials. By incorporating ethical imagery into their research outputs, faculty members can enhance the credibility and impact of their work while contributing to the broader agenda of decolonizing visual representations in academic discourse.

## Implementation at the Classroom Level

In the classroom, teaching faculty play a key role in shaping students’ educational experiences and can promote ethical image use in their courses, encouraging critical engagement with visuals and challenging dominant narratives. They influence future global health leaders who need to learn and uphold visual ethics. Students also contribute diverse perspectives to the classroom. This section outlines how faculty can integrate institutional ethical image policies into teaching and how students can help foster a culture of ethical image use.

### Review of currently used images

Teaching faculty should conduct a comprehensive review of images used in their courses to assess their alignment with ethical standards and principles. This involves critically evaluating existing images for potential harm or misrepresentation and discontinuing the use of those that perpetuate stereotypes or reinforce power differentials. By prioritizing ethical considerations in image selection, teaching faculty can create a learning environment that values diversity, inclusion, and respect for all learners. In addition, they should be prepared to engage in discussions on ethical image use if inappropriate images surface in their classroom environments.

### Incorporation of ethical image‑use discussions

Incorporating ethical image discussions into the curriculum allows the faculty to engage students in critical reflection on image creation, use, and interpretation. This can include class time for case studies and exploring sensitivity and representation issues. Faculty can facilitate open dialogue on the impact of images, guide students in assessing ethical implications, and help them navigate dilemmas in their own research and practice. Creating a safe space for this type of reflection fosters empathy, cultural humility, and empowers students to be responsible visual communicators.

### Engaging students

Engaging students in discussions and initiatives related to ethical image use provides an opportunity for institutions to foster a culture of ethical awareness and responsibility. This may include hosting seminars or workshops to introduce students to the importance of ethical imagery in global health education, incorporating ethical image‑use considerations into student orientations or training programs, and providing opportunities for student‑led initiatives to promote ethical image creation and dissemination. Many students may already have an awareness of image‑use ethics or may bring a unique perspective to the conversation, and student representatives should be included in the development of institution‑wide guidelines. By empowering students as agents of change, institutions can cultivate a sense of ownership and investment in promoting ethical practices within the academic community.

### Collaboration with community partners

Institutions should prioritize collaboration with community partners and stakeholders in initiatives related to ethical image use and creation. This may involve partnering with local organizations, institutions, or communities to co‑create images that authentically represent diverse perspectives and experiences within global health contexts. By fostering collaborative relationships based on mutual respect, reciprocity, and shared decision‑making, institutions can promote ethical image‑making practices that honor the agency and dignity of all stakeholders.

### Continuous improvement and accountability

Finally, institutions must commit to continuous improvement and accountability in their efforts to promote ethical image use and creation. This involves establishing mechanisms for ongoing evaluation and feedback, monitoring adherence to ethical standards and guidelines, and addressing any instances of non‑compliance or harm. By fostering a culture of accountability and transparency, institutions can uphold their commitment to ethical values and principles while driving meaningful change within the academic community and beyond.

## Conclusion

The intersection of decolonization and photographic imagery in global health education represents a critical frontier for advancing equity, justice, and respect for all individuals and communities. We have presented a delineation of actionable steps for universities and faculty members to transform the way they use images and thus shape narratives, perceptions, and practices within academic settings.

By upholding principles of consent, integrity, and relevance in the images we create and deem suitable for use at the university, research, and classroom levels we can challenge existing power structures, dismantle colonial legacies, and amplify marginalized voices in the field of global health. Ethical imagery serves as a powerful tool for humanizing complex health issues, fostering empathy and solidarity, and promoting inclusive and respectful engagement with diverse perspectives and experiences.

As we look towards the future, it is imperative that academia continues to prioritize the ethical imperative of decolonizing photographic imagery in global health. This entails ongoing dialogue, collaboration, and action across institutional, disciplinary, and community boundaries. By harnessing the collective efforts of educators, researchers, stakeholders, and students, we can effect sustainable change in this sphere over time, fostering a culture of ethical reflexivity and accountability to build a more just and equitable world where all individuals are valued, empowered, and dignified in the way they are portrayed visually.

## References

[r1] Khan M, Abimbola S, Aloudat T, Capobianco E, Hawkes S, Rahman‑Shepherd A. Decolonising global health in 2021: A roadmap to move from rhetoric to reform. BMJ Glob Health. 2021;6(3):e005604. doi:10.1136/bmjgh-2021-005604. PMC7993212 33758016

[r2] McCoy D, Kapilashrami A, Kumar R, Rhule E, Khosla R. Developing an agenda for the decolonization of global health. Bull World Health Organ. 2024;102(2):130–136. doi:10.2471/BLT.23.289949. 38313156 PMC10835633

[r3] Oti SO, Ncayiyana J. Decolonising global health: Where are the Southern voices? BMJ Glob Health. 2021;6(7):e006576. doi:10.1136/bmjgh-2021-006576. PMC8268905 34244206

[r4] Hirsch LA. Is it possible to decolonise global health institutions? Lancet. 2021;397(10270):189–190. doi:10.1016/S0140-6736(20)32763-X. 33453772

[r5] Farooqi R, Cardoso Pinto AM, Shariq S, Mendelson M, Charani E. Decolonising visual narratives in global health: The case for equitable and ethical imagery use. In: Shapiro L, ed. Graphic Medicine, Humanizing Healthcare and Novel Approaches in Anatomical Education. Vol 3. Biomedical Visualization. Springer Nature; 2023:41–61. doi:10.1007/978-3-031-39035-7_3.

[r6] Charani E, Shariq S, Cardoso Pinto AM, et al. The use of imagery in global health: An analysis of infectious disease documents and a framework to guide practice. Lancet Glob Health. 2023;11(1):e155–e164. doi:10.1016/S2214-109X(22)00465-X. 36463917

[r7] Bate D. The occidental tourist: Photography and colonizing vision. Afterimage. 1992;20:11–13.

[r8] Alenichev A, de Laat S, Solomon N, et al. Assembling a global health image: Ethical and pragmatic tensions through the lenses of photographers. PLOS Glob Public Health. 2024;4(2):e0002540. doi:10.1371/journal.pgph.0002540. PMC10866471 38354112

[r9] Graham AP, Lavery JV, Cook‑Deegan R. Ethics of global health photography: A focus on being more human. Health Hum Rights. 2019;21(1):49–62. 31239614 PMC6586959

[r10] Alenichev A, Peeters Grietens K, Shaffer J, et al. Global health photography behind the façade of empowerment and decolonisation. Glob Public Health. 2024;19(1):2394811. doi:10.1080/17441692.2024.2394811. 39177159

[r11] Wang C, Burris MA. Photovoice: Concept, methodology, and use for participatory needs assessment. Health Educ Behav. 1997;24(3):369–387. doi:10.1177/109019819702400309. 9158980

[r12] Sutton‑Brown CA. Photovoice: A methodological guide. Photography and Culture. 2014;7(2):169–185. doi:10.2752/175145214X13999922103165.

[r13] Cornell J, Mkhize L, Kessi S. Envisioning photovoice as decolonial feminist praxis. In: Boonzaier F, van Niekerk T, eds. Decolonial Feminist Community Psychology. Springer International Publishing; 2019:59–76. doi:10.1007/978-3-030-20001-5_5.

[r14] Desmon S. Photoshare Announces Winners of 2018 Photo Contest. Johns Hopkins University Center for Communication Programs. Published July 24, 2018. Accessed August 20, 2025. https://ccp.jhu.edu/2018/07/24/photoshare-contest-2018/.

